# Supplementary material for: Content-rich biological network constructed by mining PubMed abstracts
Source: BMC Bioinformatics. 2004 Oct 8;5:147. doi: 10.1186/1471-2105-5-147 (PMC528731; doi:10.1186/1471-2105-5-147)
Supplement: Additional File 2 — The original results of the above study (non-essential files are deleted to keep the file size under the limit set by BMC bioinformatics). [file 1471-2105-5-147-S2.bz2 › chilibotAdditionalFile2/dip05/590ID9195885E10962/html/NTHA_NTHB.html]

 


 **NTHA** and **NTHB** 
  
Found 122 abstracts in PubMed, retrieved 05.  
 

 What does Google say? 
 PDF only 
| .edu only 

---

**Interactive relationship** (e.g. stimulation, inhibition, etc)

**Non-interactive relationship** (e.g. studied together, co-existance, homology, etc.)

- ABN, AHN and AFN were converted by various microorganisms belonging to different bacterial genera e.g. Pseudomonas or Rhodococcus expressing either nitrilase  [ **NTHB** ]   [ **NTHA** ]  or nitrile hydratase activities.  Ref: 12845494 Appl Microbiol Biotechnol< MedlineTA>Appl Microbiol Biotechnol,
- In order to suppress unwanted side reactions, biotransformations were performed with recombinant Escherichia coli strains that heterologously expressed nitrilase  [ **NTHB** ]   [ **NTHA** ]  activities originating from Pseudomonas, Rhodococcus, or Synechocystis strains.  Ref: 12845494 Appl Microbiol Biotechnol< MedlineTA>Appl Microbiol Biotechnol,
- In addition, we report a rapid and convenient spectrophotometric method to monitor the activity of NI1 NHase  [ **NTHB** ]   [ **NTHA** ]  during purification.  Ref: 12729727 Protein Expr Purif, 2003
- The attempted conversion of the 2 acetoxynitriles to almost stoichiometric amounts of the corresponding 2 acetoxycarboxylic acids was finally achieved by using either a recombinant E. coli strain that highly overexpressed the nitrilase  [ **NTHB** ]   [ **NTHA** ]  gene from the pseudomonad or the purified enzyme derived from this strain.  Ref: 12845494 Appl Microbiol Biotechnol< MedlineTA>Appl Microbiol Biotechnol, 2003
- Eukaryotic NAD synthetase Qns1 contains an essential, obligate intramolecular thiol glutamine amidotransferase domain related to nitrilase  [ **NTHB** ]   [ **NTHA** ] .  Ref: 12771147 J Biol Chem< MedlineTA>J Biol Chem, 2003
- A series of iron complexes based on the pentadentate ligand bis 2 methyl 2 mercaptopropyl 1 thia diazacyclononane, bmmp TASN 2, have been synthesized and characterized as models of iron containing nitrile hydratase NHase  [ **NTHB** ]   [ **NTHA** ] .  Ref: 12844310 Inorg Chem< MedlineTA>Inorg Chem, 2003
- Here we show yeast glutamine dependent NAD synthetase Qns1 requires both the nitrilase  [ **NTHB** ]   [ **NTHA** ]  related active site residues and the NAD synthetase active site residues for function in vivo.  Ref: 12771147 J Biol Chem< MedlineTA>J Biol Chem, 2003
- Earlier we discovered that glutamine dependent NAD synthetases contain N terminal domains that are members of the nitrilase  [ **NTHB** ]   [ **NTHA** ]  superfamily and hypothesized that these domains function as glutamine amidotransferases GATs for the associated synthetases.  Ref: 12771147 J Biol Chem< MedlineTA>J Biol Chem, 2003
- These data indicate that the nitrilase  [ **NTHB** ]   [ **NTHA** ]  related domain in Qns1 is the fourth independently evolved GAT domain to have been identified in nature.  Ref: 12771147 J Biol Chem< MedlineTA>J Biol Chem, 2003
- Earlier, we noted a perfect correlation between presence of a domain related to nitrilase  [ **NTHB** ]   [ **NTHA** ]  and glutamine dependence and then proved, in an accompanying paper, that the nitrilase  [ **NTHB** ]   [ **NTHA** ]  related domain is an essential, obligate intramolecular, thiol dependent glutamine amidotransferase in the yeast NAD synthetase, Qns1.  Ref: 12777395 J Biol Chem< MedlineTA>J Biol Chem, 2003
- We report a novel heterologous system for the expression of catalytically active iron containing NI1 NHase  [ **NTHB** ]   [ **NTHA** ]  in Escherichia coli, involving coexpression with the E. coli GroES and GroEL chaperones.  Ref: 12729727 Protein Expr Purif, 2003
